# Survey-based Evaluation of Resident and Attending Financial Literacy

**DOI:** 10.5811/westjem.2021.8.53016

**Published:** 2021-11-05

**Authors:** Ryan M. Huebinger, Rahat Hussain, Keegan Tupchong, Shabana Walia, Hilary Fairbrother, Jonathan Rogg

**Affiliations:** * McGovern Medical School at The University of Texas Health Science Center at Houston (UTHealth), Department of Emergency Medicine, Houston, Texas; † McGovern Medical School at The University of Texas Health Science Center at Houston (UTHealth), Department of Internal Medicine, Houston, Texas

## Abstract

**Introduction:**

Physician finances are linked to wellness and burnout. However, few physicians receive financial management education. We sought to determine the financial literacy and educational need of attending and resident physician at an academic emergency medicine (EM) residency.

**Methods:**

We performed a cross-sectional, survey study at an academic EM residency. We devised a 49-question survey with four major domains: demographics (16 questions); Likert-scale questions evaluating value placed on personal finances (3 questions); Likert-scale questions evaluating perceived financial literacy (11 questions); and a financial literacy test based on previously developed and widely used financial literacy questions (19 questions). We administered the survey to EM attendings and residents. We analyzed the data using descriptive statistics and compared attending and resident test question responses.

**Results:**

A total of 44 residents and 24 attendings responded to the survey. Few (9.0% of residents, 12.5% of attendings) reported prior formal financial education. However, most respondents (70.5% of residents and 79.2% of attendings) participated in financial self-learning. On a five-point Likert scale (not at all important: very important), respondents felt that financial independence (4.7 ± 0.8) and their finances (4.7±0.8) were important for their well-being. Additionally, they valued being prepared for retirement (4.7±0.9). Regarding perceived financial literacy (very uncomfortable: very comfortable), respondents had the lowest comfort level with investing in the stock market (2.7±1.5), applying for a mortgage (2.8±1.6), and managing their retirement (3.0±1.4). Residents scored significantly lower than attendings on the financial literacy test (70.8% vs 79.6%, P<0.01), and residents scored lower on questions pertaining to investment (78.8% v 88.9%, P<0.01) and insurance and taxes (47.0% v 70.8%, P<0.01). Overall, respondents scored lower on questions about retirement (58.8%, P<0.01) and insurance and taxes (54.7%, P<0.01).

**Conclusion:**

Emergency physicians’ value of financial literacy exceeded confidence in financial literacy, and residents reported poorer confidence than attendings. We identified deficiencies in emergency physicians’ financial literacy for retirement, insurance, and taxes.

## INTRODUCTION

Personal finances are important for physician wellbeing and success, and between increasing education debt and stagnating reimbursements, sound financial management is becoming increasingly important for physicians.^1^–^3^ Compounding the worsening financial situation for emergency physicians (EP), the Centers for Medicare & Medicaid plan to decrease Medicare total allowable emergency medicine (EM) charges by 6% in the future,^3^ and due to the rapid opening of EM residencies, all EM jobs are projected to be filled and possibly exceeded in the coming years.^4^–^7^ These factors could impact EP salaries and their ability to manage increasing debt.

These trends are concerning as debt negatively impacts physician career choices,^8^,^9^ career satisfaction,^10^ and quality of life.^8^ Debt is also linked to depressive symptoms, cynicism,^11^ and burnout, irrespective of specialty or level or training.^12^–^15^ While there are many contributors to burnout, having a financial plan and enough money are protective of burnout.^16^ Despite the importance of personal finances to physician success and wellbeing, formal financial education has traditionally been left out of medical education^12^ as well as much of US primary, secondary, and post-secondary education; it is required for high school students in only 17 states.^17^ Emergency medicine residents also receive limited education on debt management, and many young attendings feel unprepared to manage their finances.

While there is a growing body of literature on physician wellness and burnout and despite the growing importance of financial literacy for physicians, research on financial literacy is limited, particularly for EPs. We sought to characterize perceptions of financial education as well financial literacy of residents and attendings at an academic EM residency.

## METHODS

### Study Design

We developed a financial literacy survey (Qualtrics LLC, Provo, UT), which consisted of 49 questions with four domains: demographics (16 questions); Likert-scale questions (1:5, not at all important: very important) evaluating perceived importance of personal finances (three questions); Likert-scale questions (1:5, very uncomfortable: very comfortable) evaluating self-perception of financial literacy (11 questions); and a financial literacy test based on previously developed and widely used financial literacy questions.^20^,^21^ We expanded the test to evaluate domains important to physicians (19 questions; budgeting, investment, retirement, and insurance and taxes) (Appendix 1). Using the department listserv, we distributed the survey to all current residents and faculty in the emergency department. Participants were recruited over a two-month period, from mid-August to mid-October 2019. This study was approved by the institutional review board.

### Study Population

The study took place in a three-year, urban, academic residency with 60 resident and 50 full-time faculty members. All residents and full-time faculty were invited to take part in the survey. Participation was voluntary and not incentivized.

### Study Variables and Outcomes

Respondent characteristics included age, gender, attending vs resident, years in practice, retirement account from prior career, career prior to medicine, prior formal financial education, timing of formal financial education, participation in self-financial education, and source of self-financial education. Financial characteristics included current education debt, ability to pay off their credit card, and ability to afford a $400 emergency. Outcomes were defined as overall performance on the financial literacy test as well as performance in each of the four subcategories: budgeting (four questions); investment (nine questions); retirement (three questions); and insurance and taxes (three questions).

### Statistical Analysis

We first described baseline characteristics and financial characteristics of survey respondents, comparing characteristics between residents and attendings using chi-squared, t-tests, and Wilcoxon rank-sum tests. For “the perceived importance of personal finances” section, we calculated and compared the average Likert score for each question using t-tests. We stratified perceived financial literacy into four categories: budgeting (two questions); investment (one question); retirement (two questions); and insurance and taxes (six questions). We then compared the average Likert score between residents and attendings overall and for each subsection. Lastly, we compared residents and attendings overall and subsection scores on the financial literacy test using t-tests. All analyses were performed using Stata 15.1 (Stata Corp, College Station, TX).

## RESULTS

A majority (73.3%, 44/60) of residents and half (48%, 24/50) of attendings in the department responded to the survey. The median age was lower for residents than attendings (28 vs 38.5; *P*<0.01). Gender did not differ significantly between the resident and attending groups. A minority of residents (22.7%) and attendings (16.7%) had a career prior to medicine, and while not significant, more residents had a retirement account in their prior career (50% vs 25%, *P* = 0.4). There was a similar rate of formal financial education between residents and attendings (9.1% vs 12.5%, *P* = 0.7). A majority of both residents (70.5%) and attendings (79.2%) participated in financial self-education, but there was not a significant difference between the groups (*P* = 0.4). Two residents reported having formal financial education during residency. These residents possibly pursued financial education on their own, as a financial curriculum was not available through the residency at the time of this survey (Table 1).

Education debt differed between residents and attendings, with 50.4% of residents having over $200,000 of education debt and only 12.5% of attendings having greater than $200,000 of education debt (*P* = 0.02). Residents were also more likely to have credit card debt that they could not afford to pay off (34.1% vs 8.3%, *P*<0.01). While not significant, fewer residents could afford to pay for a $400 dollar emergency (86.4% vs 100%, *P* = 0.06) (Appendix 2).

Both residents and attendings rated the importance of personal finance highly (4.54 vs 4.87, *P* = 0.1), but overall importance of finances was significantly higher than average perceived financial literacy (4.7 vs 3.4, *P*<0.01). Residents rated their confidence in all financial literacy categories lower than attendings: budgeting (3.3 v 4.6; *P*<0.01); investment (2.3 vs 4, *P*<0.01); retirement (2.7 vs 3.8, *P*<0.01); and taxes and insurance (2.6 vs 3.8, *P*<0.01) (Appendix 3).

**Figure 1:**
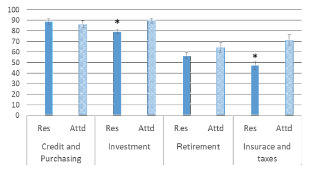
Average number of questions answered correctly on financial literacy test, stratified by topic and level of training. *P<0.01 *Res*, resident; *Attd*, attending.

Overall, residents answered fewer questions correctly than attendings on the financial literacy test (13.5, 70.8% vs 15.1, 79.5%, *P*<0.01) (Figure 3). Stratified by subsection, participants scored higher on budgeting (80.2%) and investment (82.1%) than retirement (58.8%, *P*<0.01) and insurance and taxes (54.7%, *P*<0.01). Compared to attendings, residents answered a lower percentage correctly on two of the four sections of the financial literacy test: investment (78.8% vs 88.9%, *P*<0.01) and insurance and taxes (47.0% v 70.8%, *P*<0.01). Residents and attendings scored similarly on the budgeting section (88.2% v 85.7%, *P* = 0.4) and the retirement section (56.1% vs 63.9%, *P* = 0.2) (Figure 1).

## DISCUSSION

Physician finances are linked to burnout, career satisfaction, and successful retirement. We sought to evaluate financial literacy at an EM residency. We found that prior formal financial education was uncommon (10.3%), but most participants participated in self-education (73.5%). While financial literacy was perceived as important, perceived financial literacy confidence was lower. While attendings performed better than residents overall, particularly on the investment and insurance and taxes sections, both residents and attendings struggled with questions about retirement and insurance and taxes.

Unfortunately, we have little understanding of the financial literacy of physicians. Limited prior studies evaluating financial literacy have found that while there is a high level of interest in financial knowledge, self-perceived financial literacy is felt to be poor.^8^,^12^,^19^ We also found that while there was a very low rate of formal education, there was significant interest in financial education through self-education. Also, many residents do not feel prepared to make financial decisions as attendings, and many program directors are concerned over resident readiness to make financial decisions.^22^

The resolution for this significant problem is financial literacy education, but research on this topic is limited. Two studies have evaluated the effect of a financial education intervention and assessed investment literacy of residents finding that financial literacy was near the average for the general population, a level considered inadequate by investors.^23^ One course was very well received, with all participants strongly supporting its importance in graduate medical education.^24^ Our respondents similarly highly valued financial knowledge.

We identified key deficiencies in financial education for residents and attendings, particularly retirement, insurance, and taxes. While financial literacy was highly valued, residents and attendings lacked confidence. Further studies should include other residencies to improve the validity of our results and better characterize financial education needs for EPs. Given the importance of financial literacy in addition to lack of standardized financial literacy curriculums, residencies should prioritize development of financial literacy curriculums, with particular emphasis on retirement, insurance, and taxes.

## LIMITATIONS

We conducted the study at a single institution with a small number of participants. Future studies should include other institutions to improve generalizability. The recruitment for the survey was voluntary, and attending participation was somewhat limited, which could have led to selection bias. Respondents may have been hesitant to report their inability to afford a $400 dollar emergency, leading to response bias for this question. Using the categorical Likert scale leads to results that are subjective and have limited external comparability. However, this did allow us to compare perceived importance of finances to perceived financial literacy. Our financial literacy test was based on expert-developed financial literacy tests, but experts recognized that financial literacy is difficult to evaluate.^25^ We attempted to best capture what we felt were the most important financial literacy domains for physicians, but this is ultimately subjective in nature.

## CONCLUSION

Emergency physicians’ value of financial literacy exceeded confidence in financial literacy, and residents reported poorer confidence than attendings. We identified deficiencies in emergency physicians’ financial literacy for retirement, insurance, and taxes.

This research was presented at the 2020 Society of Academic Emergency Medicine Meeting.

## Supplementary Information



## Figures and Tables

**Table 1:** Characteristics of survey respondents, stratified by level of training.

	Residents (n = 44)	Attendings (n = 24)
Age; median (IQR) (P<0.01)	28 (27–29.5)	38.5 (35–43)
Female	9 (37.5%)	14 (31.8%)
Had a career prior to medicine	10 (22.7%)	4 (16.7%)
Years in prior career; median (IQR) (P<0.01)	4.5 (1.5–5)	4 (3–5)
Had a retirement account in their prior career	5 (50.0%)	1 (25.0%)
Had received formal financial education	4 (9.1%)	3 (12.5%)
Where formal financial education was obtained		
Undergraduate	1/4 (25.0%)	0/3 (0.0%)
Medical school	0/4 (0.0%)	2/3 (66.7%)
Residency	2/4 (50.0%)*	1/3 (33.3%)
Other	1/4 (25.0%)	0/3 (0.0%)
Participate in finance self-education	31 (70.5%)	19 (79.2%)
Source of finance self-education		
Books	19/31 (61.3%)	14/19(73.7%)
Website	25/31 (80.7%)	18/19 (94.7%)
Podcasts	12/31 (38.7%)	7/19 (36.8%)
Financial advisor	12/31 (38.7%)	10/19 (52.6%)
Other	2/31 (6.5%)	3/19 (15.8%)

*IQR*, interquartile range.
