# Supplementary material for: Survey-based Evaluation of Resident and Attending Financial Literacy
Source: West J Emerg Med. 2021 Nov 5;22(6):1369–73. doi: 10.5811/westjem.2021.8.53016 (PMC8597697; doi:10.5811/westjem.2021.8.53016)
Supplement: Supplementary file 1 [file wjem-22-1369-s001.docx]

**Appendix 1. Financial literacy test as it appears as part of the financial literacy survey**

Q20 True or False

|  |  | |
| --- | --- | --- |
|  | True (1) | False (2) |
| Your credit card rating is not affected by how much you charge on your card. (1) |  |  |
| If your credit car is stolen & someone uses it before you report it missing, you are only responsible for $50 no matter how much they charge on it. (2) |  |  |
| Mutal funds pay a guaranteed rate of return. (3) |  |  |
| Buying a single company stock usually provides a safer return than a stock mutual fund. (4) |  |  |
| Stocks are normally riskier than bonds. (5) |  |  |
| If the interest rate of an adjustable rate mortgage goes up, your monthly mortgage payment will also increase. (6) |  |  |
| Employers are responsible for providing the majority of your retirement funds. (7) |  |  |
| The cash value of a life insurance policy is the amount available if your surrender your life insurance policy while you are alive. (8) |  |  |
| After signing a contract to buy a new car, you have 3 days to change your mind. (9) |  |  |

Q21 What % of your retirement account could you safely spend annually in retirement?

- 1% (1)
- 3% (2)
- 6% (3)
- 10% (4)

Q22 If you invest $100 at 10% compounded annually, how much money will you have in 2 years?

- $110 (1)
- $120 (2)
- $121 (3)
- $131 (4)

Q23 If you invest $100 at 8%, approximately how many years will it take to have $200?

- 5 years (1)
- 9 years (2)
- 12 years (3)
- 15 years (4)

Q24 Which of the following has guaranteed returns?

- Stocks (1)
- Corporate Bonds (2)
- Savings Acount (3)
- Mutual Funds (4)

Q25 Which of these is typically considered the most risky investment?

- Individual Stocks (1)
- Government Bonds (2)
- Mutual Funds (3)
- A Single ETF (4)

Q26 When can money invested in a 401k typically be taken out without penalty?

- 59 ½ (1)
- 60 (2)
- 64 ½ (3)
- 65 (4)

Q27 Which of these is not an insurance doctors commonly have?

- Tail (1)
- Umbrella (2)
- Essential Employee (3)
- Disability (4)

Q28 Assume you make $100k and pay a total of $20k in taxes. On the last $10k of earnings, you pay $5k in taxes. What is your marginal tax rate?

- 5% (1)
- 10% (2)
- 20% (3)
- 25% (4)
- 50% (5)

Q29 If the interest rate on your savings account is 1% annually & the annual inflation is 2%; after 1 year would you be able to buy:

- More than before (1)
- Exactly the same (2)
- Less than before (3)

Q30 Investing money across different assets makes the risk of losing money

- Increase (1)
- Decrease (2)
- Stay the same (3)

**Appendix 2**. Comparison of baseline financial characteristics for residents and attendings

|  | Residents (n=44) | Attendings (n=24) | p-value |
| --- | --- | --- | --- |
| Current educational debt^1^ |  |  | 0.02 |
| $0 | 8 (18.2%) | 13 (54.2%) |  |
| $1-100K | 3 (6.7%) | 5 (20.8%) |  |
| $100-200K | 11 (25.0%) | 3 (12.5%) |  |
| $200-300K | 16 (36.4%) | 3 (12.5%) |  |
| 300K-400K | 5 (11.4%) | 0 (0.0%) |  |
| 400-500K | 1 (2.7%) | 0 (0.0%) |  |
| Do you have credit card debt you do not pay off fully at the end of the month?^2^ | 15 (34.1%) | 2 (8.3%) | <0.01 |
| Can you afford a $400 emergency expense without borrowing money or using a credit card?^2^ | 38 (86.4%) | 0 (0.0%) | 0.058 |

1. Compared using a Wilcoxon ranksum test
2. Compared using Chi squared test

**Appendix 3**. Average Likert score for perceived financial literacy, stratified by topic
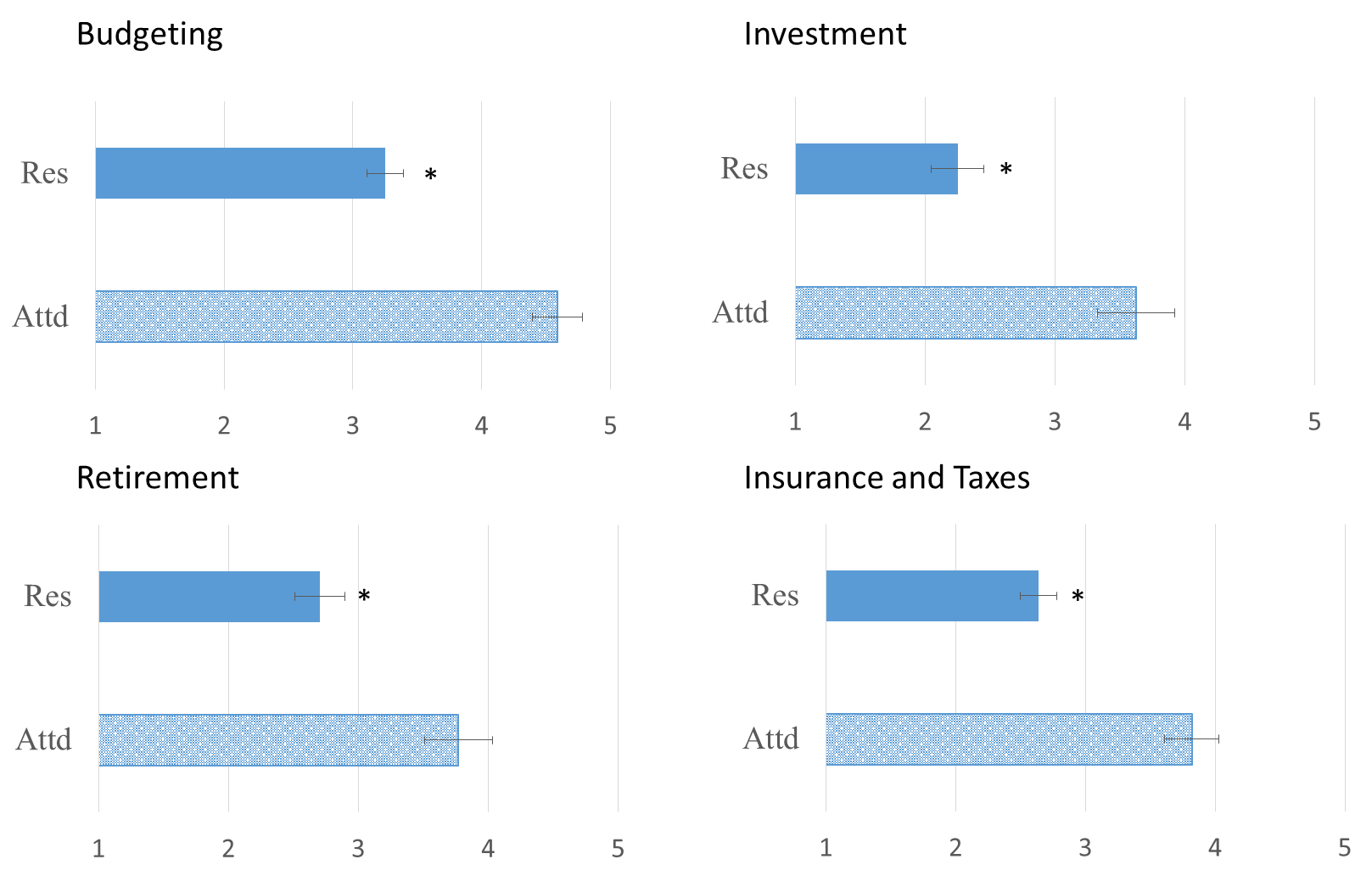


1. * p<0.01
2. Res – Resident; Attd – Attending
